# Emotional Noun Processing: An ERP Study with Rapid Serial Visual Presentation

**DOI:** 10.1371/journal.pone.0118924

**Published:** 2015-03-04

**Authors:** Shengnan Yi, Weiqi He, Lei Zhan, Zhengyang Qi, Chuanlin Zhu, Wenbo Luo, Hong Li

**Affiliations:** 1 School of Psychology, Liaoning Normal University, Dalian, China; 2 Laboratory of Cognition and Mental Health, Chongqing University of Arts and Sciences, Chongqing, China; 3 Research Centre of Brain Function and Psychological Science, Shenzhen University, Shenzhen, China; Zhejiang Key Laborotory for Research in Assesment of Cognitive Impairments, CHINA

## Abstract

Reading is an important part of our daily life, and rapid responses to emotional words have received a great deal of research interest. Our study employed rapid serial visual presentation to detect the time course of emotional noun processing using event-related potentials. We performed a dual-task experiment, where subjects were required to judge whether a given number was odd or even, and the category into which each emotional noun fit. In terms of P1, we found that there was no negativity bias for emotional nouns. However, emotional nouns elicited larger amplitudes in the N170 component in the left hemisphere than did neutral nouns. This finding indicated that in later processing stages, emotional words can be discriminated from neutral words. Furthermore, positive, negative, and neutral words were different from each other in the late positive complex, indicating that in the third stage, even different emotions can be discerned. Thus, our results indicate that in a three-stage model the latter two stages are more stable and universal.

## Introduction

We experience a range of emotions in our everyday life. These experiences can be as varied as being overwhelmingly happy to indescribably sad. As emotion is so closely related to our daily life, it is not surprising that affective processing in the human brain has received so much interest. Thus far, there is a general consensus on there being two dimensions of affect in emotion. One dimension, emotional valence, describes the extent to which an affective response is pleasant or unpleasant, and therefore elicits an appetitive or aversive response [1]. The other dimension is arousal, the level of activation associated with an emotion, and is described as being exciting or calming [1–3]. With the development of the event-related potential (ERP) approach, more and more researchers have focused on the time course of affective processing, and have employed emotional pictures, facial expressions, and emotional words as stimuli to elicit affective responses. Emotional words are typically used in human communication, and research on these words is important.

Several studies have indicated that the presentation of emotional words might affect ERP components as early as the P1, P2, and N1 [4–7]. Van Hooff et al. (2008) showed that the P1 is significantly associated with the correct detection of facial expressions, and researchers have speculated that this component might represent the detection of a facial emotion, albeit without the ability to distinguish different types of emotions. In an emotional Stroop task, negative words elicited larger P1 amplitudes than neutral words [8]. On the other hand, N170 is a face-sensitive component that can be modulated by expression, with emotional facial expressions eliciting larger N170 amplitudes than neutral facial expressions [9]. However, some studies have found that emotional words can elicit a similar N170 component [10,11].

Besides these two components, it has been shown that a late ERP component, namely the late positive complex (LPC), can be evoked by emotional stimuli. This component occurs approximately 400 ms after a stimulus, and belongs to the P3 family of ERP components. When emotional stimuli are presented, the activation of this component has been observed at central-parietal electrode sites [12,13]. Fischler and Bradley (2006) reported that the emotional effect of LPC components occurs when a task is related with affect, and that this does not occur in a lexical decision task (LDT). Thus, the effect of the LPC could just be related with the task rather than emotional category [14]. However, studies of emotional word processing have revealed enhanced LPC amplitudes for both positive and negative words, as compared to neutral words [15,16]. Furthermore, different LPC amplitudes can be elicited in response to positive and negative words [17–19]. These studies suggest that the valence of emotion can be discriminated in the LPC.

In 2010, Luo et al. employed the rapid serial visual presentation (RSVP) task to detect the time course of facial expression processing. In accordance with their study, they proposed a three-stage model in which the first stage could be reflected by N1 and P1. Specifically, they proposed that this first stage was when negative facial expressions could be differentially detected with negativity bias. The N170 and vertex positive potential (VPP) make up the main components of the second stage where VPP/N170 respond differently to emotional facial stimuli than neutral facial stimuli. Finally, in the third stage, positive or negative facial expressions could be separated with N300 and P3 components likely reflecting further evaluation of the affective valence of stimuli during this stage. Previous work found that facial expression processing could be characterized by the three stages only in the dual-target and short lag (stimulus onset asynchrony (SOA = 238 ms) conditions [20]. Moreover, Zhang et al. (2013) employed single-trail analysis to predict facial expression categories in individual trials with the three-stage model, and their results indicated the predictive power of the model during that task [21].

Facial expressions, emotional words, and pictures are all ways to elicit emotional processes. Previous studies on facial expressions and pictures seem to show that pictorial stimuli elicit stronger emotional effects than do words in the brain. Thus, words may represent a more symbolic level and, compared to pictorial stimuli, may evoke a smaller emotional effect. However, it is also possible that words contain less biological information. Recent research suggests that different stimuli, like facial expressions, pictures, and words, may have a similar effect in terms of eliciting emotions [22–24]. For example, Zhang et al. (2014) used the RSVP to detect the time course of emotional adjective processing, and found that affective processing evoked by emotional words had properties similar to the three-stage model of facial expression emotional processing. However, Zhang et al. only tested adjectives, and it could be that emotional adjectives and nouns are processed differently. It is thus an open question as to whether other types of words have a similar effect and involve the three above mentioned stages. In the present study, we chose several emotional nouns; specifically, we selected words that refer to people, things, what, when, emotions, concepts, abstract things, and so on. As adjectives are always used to modify nouns, we sought to assess whether words of perceptual intuition would still represent the three stages of emotional word processing.

In our study, we used the RSVP task to detect emotional processing in terms of differentiating timing, when attentional resources are limited. When a task requires attention and attentional resources are limited, participants have poorer performance during a dual-task compared to a single-task. This phenomenon is referred to as attentional blink (AB), and is a good way to detect whether limited attentional resources can reflect behavioral performance. Thus, using the dual-task RSVP paradigm, we assessed the following questions: 1) whether emotional nouns could influence early P1 components in the same way as observed in the previously described emotional adjective experiment, 2) whether emotional nouns can also elicit the N170 component, separate emotion from neutral words, and exhibit a left hemisphere advantage [7,10] 3) in the third stage, whether the LPC can distinguish different emotion types (i.e., positive, neutral, and negative).

## Methods

### Subjects

Seventeen undergraduates (12 females) ranging between 18–27-years-old (mean age = 20.3 years) were selected from Liaoning Normal University in exchange for payment. All of the participants were right-handed and with normal or corrected-to-normal vision. All subjects gave written informed consent in our experiment. They were informed of their right to withdraw at any time. The study was approved by Liaoning Normal University Human Research Institutional Review Board in accordance with the Declaration of Helsinki (1991).

### Stimuli

Stimuli consisted of 24 Chinese nouns, 12 Chinese pseudowords, and four strings of four repeated digits (i.e., 1111, 2222, 5555, and 6666; more details about experimental methods are presented in the S1 Table). The pseudowords consisted of two separate words and we ensured that their combination was meaningless (S1 Fig.). Furthermore, all pseudowords were presented upside-down. Nouns were selected from the Chinese Affect Words System (CAWS, it contains 1500 two-character emotional adjectives, 1500 two-character emotional nouns and 1500 two-character emotional verbs, they all selected from Modern Chinese Dictionary of Commonly Used Words. These words were rated by 124 participants in valence, arousal, and dominance with 9-point scale.), and were comprised of three types of valence (eight positive, eight negative, and eight neutral). All three types of nouns appeared with similar frequencies [F (2, 14) = 1.32, P = 0.728; positive = 48.38 ± 18.28 (M ± SD), neutral = 62.50 ± 18.24, negative = 55.88 ± 17.68]. The strokes also had no significant difference through three emotional conditions (F (2, 14) = 1.057, P = 0.427; positive = 19.50 ±2.07 (M ± SD), neutral = 17.38 ± 2.77, negative = 19.88 ± 2.42) while they differed significantly in valence (F (2, 14) = 536.87, P < 0.001, $\eta_{p}^{2}$ = 0.987; positive = 6.77 ± 0.17, neutral = 5.19 ± 0.42, negative = 2.58 ± 0.23). We specifically sought to distinguish between the two dimensions of emotional processing, valence and arousal, by controlling arousal across positive, negative, and neutral word valence conditions [F (2, 15) < 1; positive = 5.20 ± 0.21, neutral = 5.26 ± 0.51, negative = 5.52 ± 0.57]. The font of the characters was Song Ti No.48, and all stimuli were presented as white-colored words placed in the center of a black background. All stimulus pictures had the same size of 142×74 pixels, and had a similar viewing angle of 2.4×4.5° The screen resolution was 60 pixels per inch. Subjects were seated in a sound-proof room with their eyes approximately 90 cm from a 17-in screen.

### Procedure

**Fig 1:**
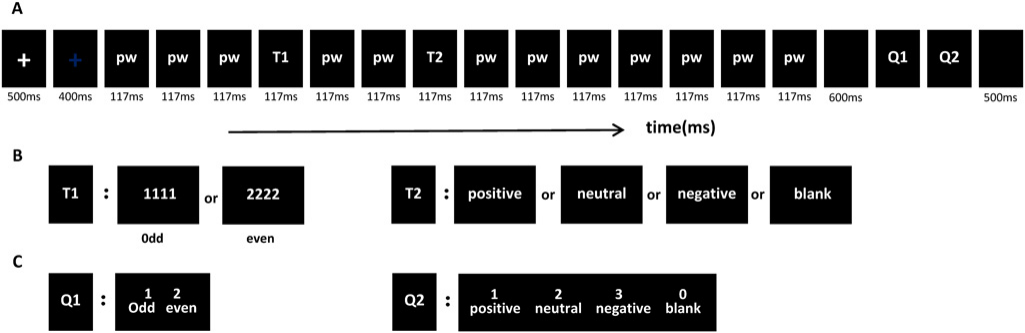
The RSVP paradigm used in this experiment. All words were in Chinese (containing pseudo-words and emotional nouns; see supplementary material for more details), and (A) each trial contained 12 pseudo-words (pw), two target stimuli (T1 and T2), and two questions (Q1 and Q2). The time interval between T1 and T2 was 234 ms. (B) Example for the two target stimuli in each trial. (C) Example for the two questions in each trail.

Similar to previous studies [20,25] we chose to employ the dual-target RSVP paradigm in the current experiment [26]. The experimental procedure was programmed with E-Prime 1.1 (Psychology Software Tools, Inc., Pittsburgh, PA). As shown in Fig. 1, each trial started with a 500-ms white fixation cross, and was followed by a 400-ms blue fixation cross. Subsequently, a sequence of stimuli were presented, each of which was visible for 117 ms. These stimuli contained 12 distracting stimuli—peseudowords, which appeared upside-down, and two target stimuli. The T1 stimulus was one of the four upright digit stings, and had an equal probability of occurring at the fourth or sixth position. The T2 stimulus was one of the three categories of upright nouns and was presented with equal probability between emotion types. To remove the superposed electrical activity elicited by the prior- and post-distractors, and to obtain ERP components elicited purely by T2, a baseline condition was designed with a blank black screen at T2 [20,27,28]. In this case, T2 occurred at the third position after T1 (lag3, SOA = 351 ms). At the T2 position, three types of nouns or a blank condition occurred; the frequency of each of these conditions was randomly and equally counterbalanced. When a sequence was presented on the screen, subjects were instructed to focus on it without any response. After a series of stimuli, subjects were required to respond to two questions regarding the parity of T1 (specifically, they were instructed to press Key “1” when the stimulus was odd and Key “2” if it was even) and the emotional category of T2 (press Key “1” if T2 was positive, Key “2” when neutral, Key “3” when negative, and Key “0” when the participant did not see T2). The picture of asking the question would disappear as the subjects pressed the index key, and this task was not assigned a specific time limit (Fig. 1). We required subjects to respond to the questions as accurately as possible.

### Electrophysiological recordings and analysis

Electrical activity of the brain was recorded (Brain Product) with FCz as a reference electrode and a 64-channel amplifier. We also employed an off-line reference to revert to the averaged reference. The vertical electrooculogram (EOG) was recorded with electrodes placed above and below the left eye. The horizontal EOG was recorded as the left versus right orbital rim. The EEG and EOG signals were amplified with a 0.01–100 Hz bandpass filter and were continuously sampled at 500 Hz/channel. Impedance was kept below 5 kΩ. The EEG data were analyzed for ERPs with the Brain Vision Analyzer software (Brain Products). ERPs were filtered with a high-frequency cutoff of 30 Hz (roll-off, 24 dB per octave) before further processing. Trials containing blinks, eye movements, or other artifacts (EEG sweeps with amplitudes exceeding ± 80 *μ*V) were excluded from averaging. Only correctly performed trials were included in the analysis.

ERP data were locked to the T2 stimuli, the images of nouns. We employed Analyzer 2.0 (Brain Product) to deal with ERPs and SPSS 16.0 to treat the exported ERP and behavioral data. The average ERP epoch was 1200 ms, and the pre-stimulus baseline was 200 ms. Our segmentation was based on emotional types, as well as the blank condition. To obtain the pure emotional effect elicited by T2 stimuli, we employed the blank condition, using this technique, we were able to observe the mean amplitudes of P1, N170, and LPC. In the P1 component, we chose six electrodes (PO3, POz, PO4, O1, Oz, O2) and a time window of 166–196 ms. In the N170 component, we chose four electrodes (P7, P8, PO7, PO8), and a time window of 264–304 ms. Finally, in the LPC component, we chose six electrodes (C3, Cz, C4, CP3, CPz, CP4) and a time window of 468–548 ms. We next used a three-way repeated measures analysis of variance (ANOVA) to analyze the mean amplitudes of the P1, N170, and LPC components. The different measures included in the ANOVA are as follows: three emotion types (positive, neutral, and negative), electrode site level (according to different components as mentioned above), and hemisphere (two levels for N170: left and right; three levels for P1 and LPC: left, middle, and right). *P* values were corrected with the Greenhouse-Geisser correction.

## Results

### Behavioral data

The ANOVA, revealed a significant main effect of emotion type (*F* (2, 32) = 10.82, *p* = 0.001, $\eta_{p}^{2}$ = 0.403). A pairwise comparison showed that the accuracy of negative words (M ± SD, 89.8 ± 14.2%) was higher than that of positive (74.5 ± 18.0%, *P* = 0.004) and neutral (68.2 ± 12.8%, *P* < 0.001) words. There was no significant difference between positive and neutral words (*p* = 0.935).

### ERP data

#### P1

According to the results of the ANOVA, positive (M ± SD, 1.92 ± 0.46 μV, *P* > 0.05) and neutral (2.11 ± 0.46 μV, *P* > 0.05) words exhibited no significant difference with negative words (1.88 ± 0.34 μV), and there was no significant difference between positive and neutral words (*P* > 0.05).

#### N170

**Fig 2:**
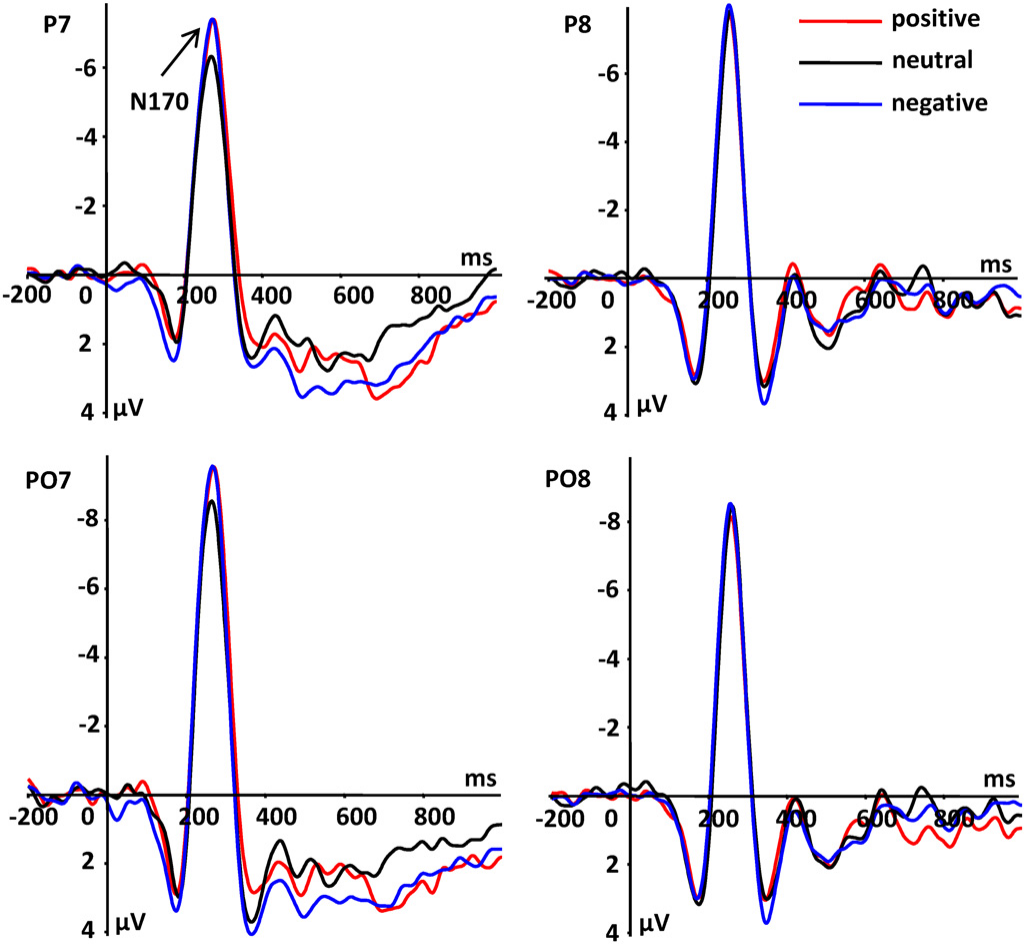
Average ERPs of N170 components at indicated electrode sites.

According to the results of the ANOVA, there was a significant main effect of hemisphere [*F*(1, 16) = 8.59, *P* = 0.010, $\eta_{p}^{2}$ = 0.349], and the left hemisphere (M ± SD, -6.88 ± 0.93 μV) elicited a larger N170 amplitude than the right hemisphere (-4.53 ± 0.60 μV). There was also a significant interaction between emotional type of noun and hemisphere [*F*(2, 32) = 10.18, *P* = 0.002, $\eta_{p}^{2}$ = 0.576]. A simple effect analysis showed that in the left hemisphere, positive (-7.47 ± 0.95 μV, *P* = 0.025) and negative (-7.09 ± 1.01 μV, *P* = 0.024) nouns elicited larger N170 amplitudes compared to neutral nouns (-6.07 ± 0.95 μV). However, in the right hemisphere, there was no significant effect between positive (-4.32 ± 0.73 μV, *P* = 0.379) and neutral (-4.73 ± 0.56 μV) nouns, and between negative (-4.54 ± 0.59 μV, *P* = 0.474) and neutral nouns (see Fig. 2).

#### LPC

**Fig 3:**
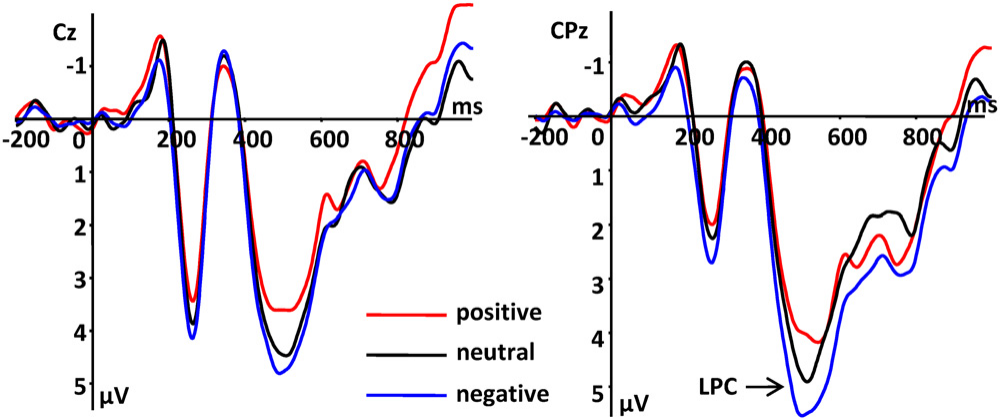
Average ERPs of LPC components at indicated electrode sites.

**Fig 4:**
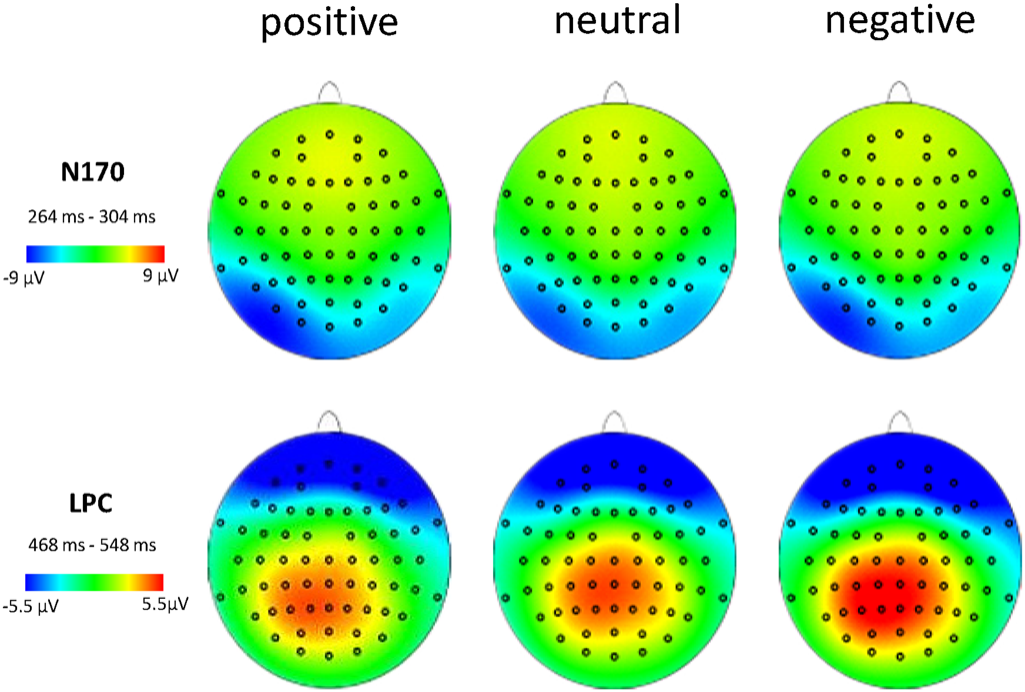
Average ERPs of N170 and LPC component topographies across three conditions.

There was a significant main effect between the type of emotional noun [*F* (2, 32) = 10.01, *P* = 0.002, $\eta_{p}^{2}$ = 0.572], electrode [*F* (1,16) = 13.86, *P* = 0.002, $\eta_{p}^{2}$ = 0.464], and hemisphere [*F*(2,32) = 8.51, *P* = 0.003, $\eta_{p}^{2}$ = 0.532] for LPC amplitudes. A pairwise comparison of emotion type indicated that negative nouns (4.28 ± 0.43 μV) compared to positive (3.16 ± 0.49 μV, *P* = 0.001) and neutral (3.71 ± 0.46 μV, *P* = 0.027) nouns elicited larger LPC amplitudes, while neutral nouns elicited larger LPC amplitudes than did positive nouns (*P* = 0.048, see Figs. 3, 4). Moreover, we found that the middle region (4.42 ± 0.59 μV, *P* = 0.002) elicited a larger LPC than the right region (2.79 ± 0.41 μV), and that there was no significant difference between right and left hemispheres (3.94 ± 0.48 μV, *P* = 0.060). We also observed no significant difference between left and middle regions (*P* = 0.530). Finally, the CPz electrode elicited the largest LPC amplitude (4.67 ± 0.55 μV, see Fig. 4).

## Discussion

In the present study, we employed a dual-target RSVP task to detect the time course of emotional word processing using ERPs. The RSVP is a good way to observe emotional word processing in terms of time, as well as the rapid response to emotional words. Moreover, this task can reflect emotional responses under conditions requiring differing amounts of attention allocation. Using this approach, we were also able to compare previous emotional valence with our results of emotional adjectives. Our behavioral data showed that accuracy rates in terms of the response to negative nouns were higher than in response to positive or neutral nouns. For ERPs, the N170 component responded differently to neutral stimuli than it did for stimuli involving positive and negative words. Moreover, different emotion types showed a separation in the LPC.

In previous studies [20,25], emotional word accuracy appeared higher for neutral words, and no difference was reported between positive and negative words. In our results, the accuracy of negative words was significantly different from positive and neutral words, but there was no significant difference between positive and neutral words. As with previous results [18,22,25], the higher accuracy of negative words compared to neutral words may be because of the influence of emotion delivery: i.e. negative emotions are stronger than neutral emotions. However, as positive and neutral words exhibited no significant difference in the current study, there was some disagreement with previous results [18,22,25]. This discrepancy may be related to the emotional words that we chose or to our different task design [19]. For example, Zhang et al. also chose emotional adjectives as stimuli with RSVP, but their adjectives always described state and characteristics. Moreover, Palazova et al. (2011) reported that, compared with emotional verbs and nouns, affective adjectives have a greater relation with emotions. Nouns, rather, usually refer to people, times, places, things, emotions, or concepts. It is possible that these different word characteristics or properties were responsible for our results. Compared to the study by Palazova et al. (2011), who employed a LDT, we used a dual-target RSVP task and focused on the emotion category. Thus, it is also possible that this different task was responsible for our findings.

According to the three-stage model for processing emotional adjectives [25], emotional affects are reflected in P1, with negative conditions eliciting larger amplitudes. P1 is generally recognized as a component under early attention allocation, and is observed in the extra-striate cortex [29]. A previous study on facial expressions [20] was generally consistent with the prior report on processing emotional adjectives, with the exception that in the latter study, the N1 component was not found in the first stage. Consistent with the findings of emotional adjectives, we observed no significant difference in the N1 component; however, we also found no significant difference in the P1 component compared to previous research. Our results may have differed from the previous studies because of our stimulus properties; namely, that facial expressions as visual stimuli are more salient and emotionally engaging than words. Additionally, there was no significant difference between negative words compared to positive and neutral words. Compared to Zhang et al.’s study, our results might be explained by the properties of nouns in comparison to adjectives. More specifically, the relation of nouns with emotion is smaller than the relation between adjectives and emotion [18]. Thus, with a reduced emotional impact from nouns, affective differences may not have developed at earlier stages of processing.

In the second stage, the difference between emotional and neutral stimuli can be distinguished, however, a finer distinction between emotional stimuli (positive and negative) has not been observed in the three-stage model of emotional adjectives [25]. Moreover, second-stage components are always lateralized toward the left for words compared to facial expressions [25,30]. The N170 component is usually recognized as a face-specific ERP component. However, some research has reported that emotional words also elicit N170 [10,11], thus, this component is a good way to judge the difference between right and left hemispheres. Consistent with the results of the previous study on emotional adjectives [25], our ERP results showed that positive and negative nouns elicited larger N170 amplitudes than did neutral nouns, and that there was an interaction between emotional condition and hemisphere. We also observed a larger N170 amplitude in the left hemisphere for emotionally weighted stimuli compared to neutral nouns. However, there was no significant difference between emotional and neutral nouns in the right hemisphere. Taken together, our results raise the question of whether findings of the first stage are inconsistent with those observed in the second stage. In other words, we attributed the lack of difference in the P1 component to the dissimilarity between emotional nouns and adjectives, while our N170 result was obtained with the same adjective/noun distinctions. We believe that this is not a problem since words with different properties can still have differential effects that are elicited simply by longer manifestation times (second rather than first stage). Indeed, in the second stage (N170 component), the emotional effect that appeared was consistent with previous studies on emotional adjectives. This indicates that the emotional effect of nouns is similar with that of adjectives, albeit only at a later stage. This result is also in agreement with the idea that word processing is almost always lateralized to the left hemisphere of the brain [7,10,11,30]. To summarize the results of our data in this time window, we suggest that the second stage was used to distinguish emotional and neutral nouns, but not between emotional nouns.

As we known that the P1 typically occurred around 100ms, and N170 around 170ms, but in our results, we analyzed P1 during 166–196ms, and N170 between 264–304ms. It also could be possible and previous studies [20,21,25,31,32] also found the delayed latencies of P1, VPP, and N170 using RSVP paradigm. Some previous studies reported different latencies for P1, VPP, and N170 components. For example, the face-specific ERP component of the intracranial recording study by Allison et al. [33] was N200; they also found a P150 component preceding the face-specific N200. Another study described a face stimulus-elicited N170 with a peak latency of ~200 ms [34]. An intracerebral recording study conducted in epileptic subjects revealed that a famous/unfamiliar face recognition task elicited an N240 component in the posterior and middle fusiform gyrus [35]. In some ERP studies [27,28] using an AB paradigm, investigators had found peak latencies for P1 persisting at ~100 ms. There were a possible methodological reasons that may explain the later P1 peak latencies in our study. We presented T1, T2 and distractive stimuli successively while prior studies with shorter latencies [27,28] separated the stimuli in time with blank screens. This factor may also explain the peak latency delays observed for N170.

Moreover, we considered the negative ERP component at occipito-temporal sites during 250–290 ms as the N170 component because the characteristics of this component were consistent with previous N170 studies [20,21,25,32]. In the previous studies, we used a very similar RSVP paradigm with emotional adjectives as stimuli and found in the time interval of about 250–290 ms a negative component at occipito-temporal region and a simultaneous positive component at froto-central region. It has been known that visual presentation of words elicit the N170 component [10,11], however, in a region more lateralized to the left hemisphere compared to that elicited by faces [11,30]. This is consistent with our finding in the current study (Fig. 2) which shows a left lateralization of N170.

Luo et al. (2010) and Zhang et al. (2014) reported that in the third stage, all emotional facial expressions and emotional adjectives containing positive, negative, and neutral valences could be distinguished from each other. The results of our study are consistent with previous results concerning the third stage, where differences between emotional valence and the neutral condition were detectable in the LPC component. However, previous studies using the three-stage model of facial expressions and emotional adjectives [20,25] only reported that different emotional types were distinguishable, but not whether any particular negative or positive bias existed in the LPC ERP. In the third stage, our results were consistent with the model. Zhang et al.’s study found that positive words elicited larger LPC amplitudes than did negative words, however, we found the opposite in that negative nouns elicited larger LPC amplitudes. This result is in disagreement with a number of studies showing that the amplitude of the LPC evoked by pleasant words was larger than that evoked by unpleasant words [4,16,36,37]. However, a previous study [38] also reported that unpleasant words elicited larger LPC amplitudes. Thus, previous studies, along with our current findings, show that the size of amplitude deflections caused by emotional valence is inconsistent. Our results can probably be explained by the fact that the third stage represents the deep processing of words and reflects emotional information. In particular, emotional processing may make individuals particularly sensitive to whether they are in bad or negative conditions.

As is mentioned above, we controlled the arousal in three types of words to detect the effect of valence, and the result also indicated that valence could independently modulate emotional word processing. The role of valence has also been emphasized in previous studies. For example, when participants were presented with emotional pictures and words, Gianotti et al. reported that information about valence was evoked before information about arousal [39]. Likewise, Bayer et al. reported that the LPC component was unaffected by arousal when valence was controlled, indicating the importance of valence in an emotion-related ERP experiment [40]. Thus, for pure valence variation, we controlled arousal in the current set of experiments.

## Conclusion

To conclude, the results of our study are similar to those of previous studies on facial expression and emotional adjective processing. We revealed a separation between emotional and neutral conditions detectable at the N170 component; specifically, we found that after 450 ms, emotion types could be separated and distinguished. This finding may indicate that in the three-stage model of emotional adjectives, only the second and third stages apply to emotional nouns.

## Supporting Information

S1 Fig Pseudowords in test. These pseudowords were presented upside-down in the experiment.
(TIFF)

S1 Table Emotional nouns in test. (DOCX)
