# Supplementary figures and images for: Emotional Noun Processing: An ERP Study with Rapid Serial Visual Presentation
Source: PLoS One. 2015 Mar 4;10(3):e0118924. doi: 10.1371/journal.pone.0118924 (PMC4349822; doi:10.1371/journal.pone.0118924)

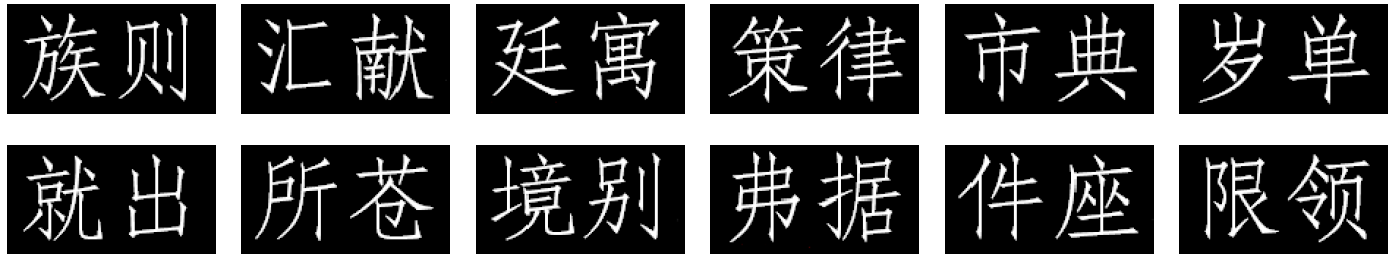

Supplement: S1 Fig — These pseudowords were presented upside-down in the experiment. (TIFF) [file pone.0118924.s001.tiff]
