# Supplementary material for: Emotional Noun Processing: An ERP Study with Rapid Serial Visual Presentation
Source: PLoS One. 2015 Mar 4;10(3):e0118924. doi: 10.1371/journal.pone.0118924 (PMC4349822; doi:10.1371/journal.pone.0118924)
Supplement: S1 Table — (DOCX) [file pone.0118924.s002.docx]

**Table S1**

Emotional nouns in test

| Positive noun | Neutral noun | Negative noun |
| --- | --- | --- |
| 好感 [good opinion]  乐趣 [joy] | 国情 [national condition]  宫廷 [palace] | 苍蝇 [fly]  耻辱 [shame] |
| 名著 [masterpiece]  诺言 [promise] | 警卫 [guard]  廉价 [cheap] | 地狱 [hell]  恶意 [malice] |
| 深情 [affectionateness]  喜剧 [comedy]  心愿 [wish]  新房 [bridal chamber] | 热泪 [hot tear]  武功 [gest]  性命 [life]  祖宗 [forefather] | 棺材 [coffin]  混蛋 [bastard]  老鼠 [mouse]  虚荣 [vanity] |
